# Factors associated with the prevalence of anterior open bite among preschool children: A population-based study in Brazil

**DOI:** 10.1590/2176-9451.19.5.103-109.oar

**Published:** 2014

**Authors:** Daniella Borges Machado, Valéria Silva Cândido Brizon, Gláucia Maria Bovi Ambrosano, Davidson Fróis Madureira, Viviane Elisângela Gomes, Ana Cristina Borges de Oliveira

**Affiliations:** 1 MSc in Dentistry, Federal University of Minas Gerais (UFMG); 2 Professor, School of Dentistry - State University of Campinas (UNICAMP); 3 PhD resident, Biological Sciences Institute of UFMG; 4 Professor, School of Dentistry - UFMG

**Keywords:** Oral health surveys, Open bite, Preschool child

## Abstract

**INTRODUCTION:**

The aim of this study was to identify factors associated with the prevalence of anterior open bite among five-year-old Brazilian children.

**METHODS:**

A cross-sectional study was undertaken using data from the National Survey of Oral Health (SB Brazil 2010). The outcome variable was anterior open bite classified as present or absent. The independent variables were classified by individual, sociodemographic and clinical factors. Data were analyzed through bivariate and multivariate analysis using SPSS statistical software (version 18.0) with a 95% level of significance.

**RESULTS:**

The prevalence of anterior open bite was 12.1%. Multivariate analysis showed that preschool children living in Southern Brazil had an increased chance of 1.8 more times of having anterior open bite (CI 95%: 1.16 - 3.02). Children identified with alterations in overjet had 14.6 times greater chances of having anterior open bite (CI 95%: 8.98 - 24.03).

**CONCLUSION:**

There was a significant association between anterior open bite and the region of Brazil where the children lived, the presence of altered overjet and the prevalence of posterior crossbite.

## Abstract (pt)

**INTRODUÇÃO:**

este estudo objetivou identificar os fatores associados à prevalência de mordida aberta anterior em crianças brasileiras com cinco anos de idade.

**MÉTODOS:**

foi realizado um estudo transversal analítico com dados do inquérito epidemiológico nacional de saúde bucal SB Brasil 2010. O desfecho estudado foi a mordida aberta, classificada em presente ou ausente. As variáveis independentes foram classificadas em individuais, sociodemográficas e clínicas. Os dados foram analisados por meio das análises bivariada e multivariada por meio do programa estatístico SPSS (versão 18.0), com nível de significância de 5%.

**RESULTADOS:**

a prevalência de mordida aberta anterior foi de 12,1% entre as crianças investigadas. Aqueles pré-escolares residentes na região Sul do Brasil apresentaram uma chance 1,8 vezes maior de serem diagnosticados com a mordida aberta anterior (IC 95%: 1,16 - 3,02). As crianças identificadas com alguma alteração de sobressaliência tiveram 14,6 vezes mais chance de pertencer ao grupo de crianças com mordida aberta (IC 95%: 8,98 - 24,03).

**CONCLUSÃO:**

verificou-se que mordida aberta anterior apresentou associação significativa com a região brasileira em que as crianças viviam, com a presença de alguma alteração de sobressaliência e com a prevalência de mordida cruzada posterior.

## INTRODUCTION

With worldwide reduction in dental caries prevalence, other oral problems have become more common.^1^^,^ 2 Malocclusion is among them and may be associated with genetic, environmental and behavioral factors, thereby resulting in morphological, functional and esthetic problems.^3^

Anterior open bite (AOB) and posterior crossbite have been identified as the most common occlusal abnormalities in primary dentition.^4^^,^ 5 AOB is characterized by lack of occlusal contact in the anterior region, while the remaining teeth are in occlusion.^6^^,^ 7 AOB is more prevalent in primary dentition, with a prevalence between 6.2% and 50.0% worldwide, varying according to the population group studied.^3^^,^ 4 ^,^ 5 ^,^ 8 ^-^ 11 This is most likely to be associated with an increase in overbite during the mixed dentition period, and the self-correcting nature of the majority of cases of anterior open bite in primary dentition.^5^^,^ 12

When non-nutritive sucking habits are no longer present in children, AOB tends to disappear.^3^^,^ 5 ^,^ 8 ^,^ 10 ^,^ 12 ^,^ 13 Góis et al^13^ showed that 70.1% of AOB present in primary dentition were self-corrected during the transition from primary to mixed dentition. Early treatment of AOB, during the primary or mixed dentition, usually reaches better results and reduces indices of relapse;^14^^,^ 15 ^,^ 16 thus, spontaneous correction of AOB during the initial stages might be, in part, result of individual's face and dentition development process.^12^^,^ 16

In this context, primary dentition directly influences the development of permanent occlusion. A number of anomalies and occlusal characteristics present in the primary dentition remain or even deteriorate in permanent dentition.^13^ It is important to advise parents that these habits should be eliminated before eruption of upper permanent incisors in order to allow further self-correction of this malocclusion.^3^^,^ 5 ^,^ 8 ^,^ 10 ^,^ 12 ^,^ 13 AOB is considered one of the most difficult occlusal abnormalities to be corrected in the permanent dentition, especially with respect to stability.^3^^-^ 10 ^,^ 12 ^-^ 20 Due to functional and esthetic abnormalities, AOB may cause negative psychosocial impact in many cases, predisposing individuals to low self-esteem, social alienation due to bullying, and behavioral disorders, with potential negative impact on their quality of life.^13^

The aim of this study was to identify factors associated with the prevalence of AOB among five-year-old children in Brazil.

## MATERIAL AND METHODS

### Study design

A cross-sectional analytical study was performed. Data from the Epidemiological Survey of the Oral Health Conditions of the Brazilian Population, known as "*SB Brasil 2010*", was used.^2^

### Ethical considerations

The Brazilian Oral Health Project was submitted to and approved by the National Council on Ethics and Human Research. An informed consent form was signed by all individuals participating in the study.^2^

### Sample population

The population of Brazil comprises approximately 190.7 million people, with 2.9 million children under the age of five.^21^

The epidemiological survey *SB Brasil 2010* assessed the oral health conditions of the Brazilian population in urban and rural areas, classifying it into different age ranges. The study surveyed 37, 519 individuals living in 26 state capitals in the Federal District and in 150 municipal districts of varying population sizes located in the countryside.^2^

The database created by this study is of public domain and freely accessible on the website of the Brazilian Ministry of Health.^2^

### Data collection

Data were collected in each participant's home. Data collection included an oral examination and a questionnaire. Dental teams comprised an examiner and an assistant who performed clinical data collection using instruments (oral mirror and periodontal probe), as recommended by the World Health Organization (WHO).^22^

The presence of AOB or any other form of malocclusion was registered using the Foster and Hamilton index (Table 1).^23^

**Table 1:** Foster and Hamilton index.

Diagnosis	Diagnostic criteria
Canine relationship	» Class I: Tip of upper canine in the same vertical plane as the distal surface of lower canine when in centric occlusion. » Class II: Tip of upper canine in anterior relationship to the distal surface of lower canine when in centric occlusion. » Class III: Tip of upper canine in posterior relationship to the distal surface of lower canine when in centric occlusion.
Overjet	Normal: Primary upper central incisor overjet ≤ 2 mm. With alteration: » Increased: Primary upper central incisor overjet > 2 mm. » Edge-to-edge: Upper and lower primary central incisors in edge-to-edge position. » Anterior crossbite: Lower primary central incisors in anterior relationship to upper primary central incisors in occlusion.
Overbite	Normal: Incisal tips of primary lower central incisors contacting the palatal surfaces of upper primary central incisors when in centric occlusion. With alteration: » Reduced: Incisal tips of primary lower central incisors not contacting the palatal or incisal surfaces of upper primary central incisors when in centric occlusion. » Anterior open bite: Incisal tips of lower primary central incisors below the level of the incisal tips of upper primary central incisors when in centric occlusion. » Deep bite: Incisal tips of lower primary central incisors touching the palate when in centric occlusion.
Posterior crossbite	Present: Upper primary molars occluding in lingual relationship with lower primary molars when in centric occlusion. Absent

Source: Adapted from Foster and Hamilton23.

### Sample calculation

A conglomerate sampling technique was used with three stratifications. The first used domains and primary sampling units: Capitals and municipal districts from the countryside, according to each macroregion. The second was a subdivision of municipal districts: 27 capitals plus 30 municipal districts from the countryside of each region of Brazil. The third used lottery to guarantee representativeness in the municipal districts, census sectors, and residences.

A maximum of 250 volunteers were assessed for anterior open bite in each one of the 172 cities in Brazil, thereby resulting in a total sample of 5,622 five-year-old children. The following parameters were used to calculate sample size: Values of z, variance, mean DEFT, acceptable margin of error, effect of design and non-reply rate. These data were taken from *SB Brasil 2003*.^1^

### Calibration

Each fieldwork team was properly trained in workshops of 20 hours (6 classes). Training was divided into phases as follows: 4 hours of theory, 2 hours of practical training, 8 hours for calibration, 2 hours of final discussion and 4 hours of fieldwork strategy. The technique of consensus was used to calculate the correlation between each examiner and the results obtained by consensus of the team. The model proposed by the WHO was used as reference. Kappa coefficient was calculated, weighted for each examiner, age-group and medical complaint with a value of 0.65 adopted as the minimal acceptable limit.^2^

### Study variables

The dependent variable was AOB. Table 2 describes the independent variables. 

**Table 2:** Independent variables and respective categories.

Independent variables	Category						
Age	State capital				Other city		
Region of Brazil	North		Northeast		Southeast	South	Midwest
Sex	Male				Female		
Family income^a^	< 250	251 - 500	501 - 1500^b^	1501 - 2500	2501 - 4500	4500 - 9500	> 9501
Tooth caries	deft = 0				deft > 1		
Need for treatment	Absent				Present		
Canine relationship	Class I			Class II		Class III	
Overjet	Normal			With alteration			
Posterior crossbite	Absent			Present			

a R$ (R$ 1,00 = US$ 0,49)

b population family income.

**Table 1:** Sample distribution according to the prevalence of anterior open bite and associated factors. (n = 5,622).

Independent variables	n (total)	Prevalence of anterior open bite		
		n (%)	Gross OR (CI 95%)	P value*
**Age**				
State capital	4,272	543 (16.6)	1	0.472
Other city	1,350	163 (13.7)	0.93 (0.77-1.13)	
**Region of Brazil**				
North	1,476	127 (9.41)	0.53 (0.41-0.69)	<0.001
Northeast	1,567	214 (15.8)	0.97 (0.77-1.22)	
Southeast	1,009	141 (16.2)	1	
South	751	152 (25.3)	1.75 (1.36-2.27)	
Midwest	819	72 (9.6)	0.55 (0.41-0.74)	
**Sex**				
Male	2,803	337 (13.6)	1	0.163
Female	2,819	369 (15.0)	1.12 (0.96-1.31)	
Family income**				
< 250^a^	270	37 (15.8)	1.05 (0.73-1.52)	0.335
251 to 500	894	97 (12.1)	0.77 (0.61-0.98)	
501 to 1,500^b^	2,917	386 (15.2)	1	
1,501 to 2,500	808	104 (14.7)	0.96 (0.76-1.22)	
2,501 to 4,500	309	43 (16.1)	1.07 (0.76-1.51)	
4,501 to 9,500	112	11 (10.8)	0.68 (0.36-1.28)	
> 9,500	48	5 (11.6)	0.73 (0.29-1.87)	
**Tooth caries**				
deft = 0	2,571	303 (13.3)	1	0.062
deft = > 1	3,051	403 (15.2)	1.16 (0.99-1.37)	
**Need for treatment of tooth caries**				
Absent	2,764	335 (13.7)	1	0.263
Present	2,858	371 (14.9)	1.10 (0.93-1.29)	
Canine relationship**				
Class I	4,308	385 (9.81)	1	< 0.001
Class II	941	228 (31.98)	4.32 (3.58-5.22)	
Class III	361	92 (34.20)	4.78 (3.64-6.28)	
Overjet**				
Normal	3,842	157 (4.26)	1	< 0.001
With alteration	138	44 (46.81)	19.78 (12.79-30.57)	
Posterior crossbite**				
Absent	1,142	194 (20.46)	1	< 0.001
Present	4,447	509 (12.93)	0.58 (0.48-0.69)	

OR: Odds ratio; CI 95%: Confidence interval

* c2 test

** missing values

a R$ (R$ 1,00 = US$ 0,49)

b population family income.

**Table 2:** Multiple logistic regression models explaining the prevalence of anterior open bite in five-year-old children in Brazil.

Categories	Adjusted OR [CI]	P value*
Southern Brazil	1.87 (1.16-3.02)	< 0.001
Overjet with alteration	14.69 (8.98-24.03)	< 0.001
Posterior crossbite present	0.62 (0.44-0.87)	0.006

OR: Odds ratio; CI 95%: Confidence interval.

### Data analysis

Data were analyzed using the Statistical Package for Social Sciences (SPSS for Windows, version 18.0, SPSS Inc, Chicago, IL, USA) software. First, bivariate data analysis was performed. Chi-square test was used to investigate the association between the dependent variable (AOB) and the independent variables (child's city of residence, region of Brazil, sex, family income, dental caries, need for treatment of dental caries, canine relationship, overjet, posterior crossbite) (P < 0.05). In order to identify the independent impact of each variable, multiple logistic regression was performed. The independent variables were inserted into logistic model on a decreasing scale according to their statistical significance (P < 0.25, stepwise backward procedure).

## RESULTS

Table 1 displays the results of bivariate analysis. The variables statistically associated with the prevalence of AOB among five-year-old children were: Region of Brazil in which the child lived, canine relationship, overjet and posterior crossbite (P < 0.001).

The results of multivariate analysis are shown in Table 2. Regardless of the other variables analyzed, five-year-old children from Southern Brazil were two times more likely to be identified with AOB than children in the Southeastern region of the country (OR = 1.87 [CI 95%: 1.16 - 3.02]). Preschool children diagnosed with alterations in overjet had 14.7 times greater chances of suffering from AOB (OR= 14.69 [CI 95%: 8.98 - 24.03]).

## DISCUSSION

The prevalence of AOB in the studied population of five-year-old children was 12.1%.^2^ However, there is considerable variation in such epidemiological data in worldwide literature (6.2 to 50.0%), even when the same regions of Brazil are compared.^3^^,^ 4 ^,^ 5 ^,^ 8 ^,^ 9 ^,^ 10 ^,^ 24 A direct comparison of the results yielded by different studies is difficult due to variation in diagnostic and classification criteria from an epidemiological perspective. Variations in study design, sample criteria and methods of analyzing results can also result in data discrepancy.

Multivariate data analysis confirmed the prevalence of AOB statistically associated with the region in which the child lived and also with the prevalence of posterior crossbite and alterations in overjet. The chances of children resident in the Southern of Brazil being diagnosed with AOB was nearly twice greater than that of children living in other regions of the country. This variation can be possibly explained by different cultural habits that may result in greater or less exposure to risk factors associated with AOB, such as time spent in breast-feeding, diet and variations in non-nutritive sucking habits in different regions of Brazil.^9^^,^ 13 ^,^ 24 These data corroborate the findings in the literature. Another study conducted in Southern Brazil also found a higher percentage of AOB in primary dentition when compared with studies undertaken in the Southeastern and Northeast regions.^3^^,^ 4 ^,^ 9 ^,^ 10

Regional, cultural and socioeconomic variations of each city should be considered and are the most probable explanation for the different prevalence of AOB found in other studies. A survey undertaken in the Southeastern of Brazil found a prevalence of AOB of 7.9% among 1,069 preschool children from Belo Horizonte,^4^ whereas in São Paulo there was a prevalence of 22.4% among 309 children.^3^ In Southern Brazil, particularly in Pelotas, 46.3% of 359 children had AOB in primary dentition.^19^ In the Northeastern Brazil, particularly in Recife, 30.2% of 1,308 five-year-old children had AOB.^10^ Moreover, studies outside Brazil also demonstrate a range of different results, with a prevalence of AOB among preschool children varying from 13.0% in Italy to 50.0% in Sweden.^5^^,^ 8 In addition, racial characteristics may influence the occurrence of AOB. Thus, there was significant difference in the prevalence of malocclusion between Caucasian and Afro American children aged from 3 to 5 years old, with no differences between males and females.^19^ In the present study, the statistical significance found between prevalence of AOB and the region of children's residence can also be related to diverse racial, economic and sociodemographic characteristics in Brazil. The Brazilian population is one of the most diverse in the world, with bi or trihybrid miscegenation prevailing in some regions. The country is of continental extension; thus, its population reveals great complexity and diversity, especially in terms of physical and cultural characteristics. Although the present study did not investigate the racial composition of the Brazilian population, the Brazilian Census of 2010 demonstrates that racial characteristics, which were self-declared, among children between 0-14 years old considerably vary according to each region of Brazil.^21^ The Brazilian Census of 2010 also demonstrates that higher median income and lower illiteracy indices were seen in Midwestern, Southeastern and Southern Brazil, while lower median income and higher illiteracy indices were present in Northern and Northeastern Brazil.^21^ However, family income did not influence the occurrence of AOB. Therefore, differences in race and sociodemographic characteristics may influence the prevalence of malocclusion among the population.^24^

Preschool children identified with alterations in overjet (increased edge-to-edge bite or anterior crossbite) had greater chances of having AOB.^5^^,^ 23 ^-^ 26 Non-nutritive sucking habits and tongue posture are included as environmental factors.^4^^,^ 5 Such transversal and sagittal abnormalities, which share the same etiological factors, may be associated with AOB. Considering that AOB is directly related to non-nutritive sucking habits, the increased prevalence of malocclusion at a younger age can be associated with an increased incidence of this habit among younger children. A longitudinal study of 386 children (aged 3 years old at study onset and examined again at 7 years of age) performed in Sweden found that the prevalence of non-nutritive sucking habits decreased from 66.0% to 4.0% between 3 and 7 years of age, which might have influenced the reduction of AOB incidence from 50% to 10% at the age of seven.^5^ In addition, oral respiration may also significantly contribute to the etiology of dentofacial abnormalities in children during growth.^28^ Furthermore, a study of schoolchildren from Lithuania aged between 7 and 15 years old found a significant association between nasal obstruction and increased overjet, open bite and maxillary growth.^27^ A study performed among preschool children in Brazil showed that children who had the habit of sucking a pacifier after two years of age and those who were oral breathers had a greater chance of developing malocclusion.^19^ While the design of the present study is robust, some limitations should be observed. Data assessed the presence or absence of AOB without differentiating its extension, severity and dental or skeletal impairment. Other factors such as the presence of harmful habits, facial and respiratory patterns, which are etiological factors of this malocclusion, were not investigated either. This is most probably due to the comprehensive character of the other variables studied, as well as the need for collecting brief data because of the large sample comprising 5.622 children. Data provided, however, is an accurate indicator of the prevalence of AOB in the different regions of Brazil. Such data are important for the strategic planning of government programs aimed at prevention, interception and treatment of AOB.

The present study alerts oral health care programs to the need for preventive measures that can deter or at least reduce the prevalence of this and other malocclusions among the infant population. In Brazil, the road towards an universal dental care for the general population, especially infants, is long. Orthodontic treatment is not just a matter of vanity. The more severe the problem, the greater the functional and psychological impact of anterior open bite. Child may often become target of bullying which can result in behavioral disorders and personality maladjustments. Additional studies are needed to clarify the etiology and severity of AOB according to each region of Brazil.

## CONCLUSION

Children living in Southern Brazil showed greater chances of being diagnosed with anterior open bite.

Children identified with alterations in overjet showed greater chances of having anterior open bite.
